# Individual risk assessment of adverse pregnancy outcome by multivariate regression analysis may serve as basis for drug intervention studies: retrospective analysis of 426 high-risk patients including ethical aspects

**DOI:** 10.1007/s00404-013-2723-1

**Published:** 2013-02-07

**Authors:** Rolf Becker, Thomas Keller, Holger Kiesewetter, Heiner Fangerau, Uta Bittner

**Affiliations:** 1 Center for Prenatal Diagnosis and Human Genetics, Free University of Berlin, Kurfuerstendamm 199, 10719 Berlin, Germany; 2 Acomed Statistics, Fockestr. 57, 04275 Leipzig, Germany; 3 Haemostaseologicum, Mohrenstr.6, 10117 Berlin, Germany; 4 Institute of the History, Theory and Ethics of Medicine, University of Ulm, Frauensteige 6, 89075 Ulm, Germany

**Keywords:** Low molecular weight heparin, ASS, Adverse pregnancy outcome, Preeclampsia

## Abstract

**Objective:**

To identify patients at very high risk for adverse pregnancy outcome (APO) at the 20- to 23-week scan and to assess the effectiveness of Aspirin (ASS) and low molecular weight heparin (LMWH) starting after this examination.

**Patients and methods:**

By applying an algorithm based on multivariate logistic regression analysis using the parameters maternal age, parity, body mass index (BMI), mean pulsatility index of both uterine arteries (meanPI), presence of uni- or bilateral notch, and depth of notch (mean notch index (meanNI), we retrospectively calculated the individual risk for APO of 21,302 singleton pregnancies. We isolated a subgroup of 426 patients with the highest calculated probability for APO (cpAPO > 27.8 %). 147 had been treated with ASS; 73 with LMWH, 15 patients with a combination of ASS and LMWH, and 191 patients had not received anticoagulants.

**Results:**

Administration of ASS starting after 20 gestational weeks in comparison to non-treated patients significantly reduced the frequency of intrauterine/neonatal death (IUD/NND), preeclampsia <33 weeks (PE < 33), and preterm delivery <33 weeks (PD < 33), while the frequency of IUGR showed a tendency to be elevated (*P* = 0.061). The subgroup of high-risk patients treated with LMWH was characterised by a higher a priori risk for APO and showed no significant reduction of any form of APO but an increased frequency of PE.

**Conclusion:**

Individual assessment of risk for APO by applying a simple algorithm based on biometrical/biographical as well as sonographic parameters may serve as basis for drug intervention studies. The administration of ASS in high-risk patients starting after 20 gestational weeks reduced the frequency of most of the severe forms of adverse pregnancy outcome in high-risk patients. A complication-reducing effect of LMWH starting after 20 weeks of gestation in patients could not be proven. From an ethical point of view, it may not be justified any more to preclude high-risk patients from administration of ASS or to perform studies of ASS against placebo.

## Introduction

Preeclampsia (PE), intrauterine growth restriction (IUGR), placental abruption, intrauterine or neonatal death (IUD/NND), and preterm delivery (PD) are important contributors to both perinatal and maternal morbidity and mortality [1–3]. Meanwhile, it is well known that at least part of these forms of adverse pregnancy outcome (APO), which mostly find their manifestation in the third trimester of pregnancy, have their origin in pathological trophoblast invasion which occurs in the first trimester of pregnancy [2, 4, 5]. More than 25 years ago, it could be shown that assessment of uterine artery Doppler waveforms (assessment of impedance as well as presence of notch) in the second trimester [6, 7] could predict some of the cases of APO. Quantification of the depth of notch was not performed. Meanwhile, the identification of high-risk patients has been improved by the introduction of other parameters like patient history (e.g., APO in prior pregnancy), biophysical parameters like elevated maternal age, elevated maternal body mass index (BMI) [8] or pre-existing maternal hypertension. Combination with certain serum markers gave additional information on the probability of APO [9–17]. Also the presence of thrombophilia is in discussion to be a contributing factor [2, 18–24].

Important consequence of the identification of a high-risk situation for APO in the first trimester is the administration of anticoagulants. The use of ASS to prevent APO had been introduced by Wallenburg [25] and later was shown to be effective in many studies [26–29] and several meta-analyses [30–37]. It is well known that up to now the effect of pharmaceutical interventions cannot yet be a complete prevention but a reduction of frequency and intensity of complications [12, 16, 38–40]. Recent research shows that this effect for ASS is higher if administered in high-risk patients before 16 weeks in comparison to later administration [35–37]. Also other drugs like low molecular weight heparin (LMWH) were shown to be possibly effective in the prevention of APO [41–44].

At the moment, we are far away from a situation where every woman has access to a first trimester risk assessment of APO including additional cost producing assessment of serum parameters [45]. Therefore, we still have to be prepared to a situation when we have to assess the probability of APO and identify patients at high risk for APO for the first time in the second trimester. It is unclear how effective ASS and LMWH are in high-risk patients if their administration starts after 20 weeks.

In prior studies, we had shown that depth of notch is an easily determinable parameter that gives important additional information for risk assessment of APO [46, 47]. We had developed an algorithm to quantify the probability for APO based on a combination of biographical (parity), biometrical (BMI) and the sonographic parameters impedance of uterine arteries as well as presence and depth of notch—a risk assessment for APO which can be performed easily and fast without additional laboratory cost as part of the second trimester anomaly scan [3].

The aim of this retrospective evaluation was to find out whether patients being identified by our algorithm at the 20 + 0- to 23 + 6-week scan to be at very high risk for APO did have benefit of administration of ASS and LMWH starting at this time in comparison to non-treated patients.

## Patients and methods

Covering a time span of 17 years (January 1994–December 2010), a total of 22,349 patients had been examined fulfilling the following criteria:

- singleton pregnancy from 20 + 0 to 23 + 6 gestational weeks
- viable foetus without major malformation detected during pregnancy of within the first 10 neonatal days
- duration of pregnancy more than 24 weeks
- well-known parameters maternal age, BMI and parity (questioned at the time of ultrasound)
- well-known results of Doppler sonography of both uterine arteries pulsatility index (PI) and notch index (NI)

The vast majority of the patients was referred to our center for a routine second trimester anomaly scan. The outcome of 21,302 of these pregnancies (95.3 %) was well known including information on duration of pregnancy and occurrence of any form of adverse pregnancy outcome (APO) mentioned below. In 10,365 cases, a feedback form which the patients had received at the end of the examination was returned to us. For further 10,937 cases, women were contacted by telephone by the staff of our office starting 4 weeks after the estimated date of delivery. The staffs were not informed on the result of the ultrasound examination at the time of contact.

The major part of this study group had been evaluated before [3] with the difference of having excluded all patients having been treated with anticoagulants. With this prior evaluation, we had created and validated a multivariate approach for the prediction of adverse pregnancy outcome (APO) defined as

- PD < 33 gestational weeks (PD < 33),
- IUGR (defined as a birth weight equal to or smaller than the 5th centile of sex-adjusted birth weight according to a German control group [48],
- PE defined as proteinuric pregnancy induced hypertension with two recordings of diastolic blood pressure of >90 mm Hg and proteinuria of >300 mg/24 h or ++ or more on dipstick testing,
- PA (based on the clinical symptom of severe bleeding leading to the clinical consequence of emergency delivery and assessment by the physician in charge as placental abruption),
- IUD/NND,

and used following parameters:

- maternal age,
- parity,
- BMI,
- presence of notch (no, unilateral, bilateral),
- mean of Pulsatility Index (PI) of right and left uterine artery (meanPI), and
- mean of Notch-Index (NI) of right and left uterine artery (meanNI) [3, 44].

In the present retrospective evaluation, we additionally included a total number of 630 patients who had been treated with anticoagulants: 389 with ASS (100 mg), 197 with LMWH (5.000 IU/d) and 44 with a combination of ASS and LMWH. 235 of these 630 patients received anticoagulants following the 20 + 0- to 23 + 6-week scan because they were believed to be at high risk for APO based on the parameters available at this time (high impedance of uterine arteries, presence of notch). The other 395 patients undergoing anticoagulant therapy were not at high risk for APO and had received medication beginning at earlier stages of pregnancy for reasons deriving of problems of internal medicine (for example history of thrombosis) or because of a history of multiple abortions.

Out of these 21,302 patients, those with a risk exceeding the 98th centile (27.8 %) for calculated probability of APO (cpAPO) (*n* = 426) were selected for further evaluation and formed a subgroup of 426 high-risk patients. Evaluation was performed with SAS 9.2 (SAS Institute Inc., Cary, NC, USA).

Statistical tests were applied for an exploratory analysis without correction for multiple testing. Contingency tables were analyzed with *χ* ^2^ test (in case of at one or more expected frequencies <5 with Fisher’s exact test). LOESS regression was used in terms of a smoothing technique to visualise dependency of fraction of complications on cpAPO.

## Results

**Fig. 1:**
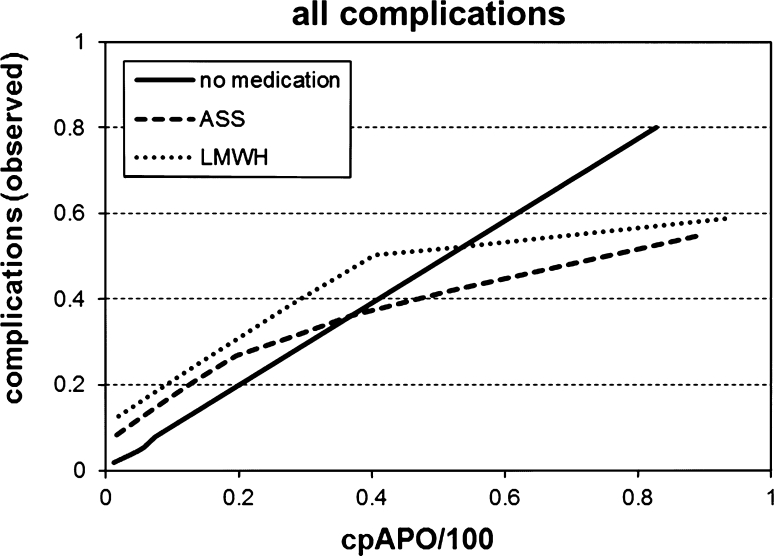
Relation of observed and calculated complications depending on mode of anticoagulation visualized by LOESS—regression analysis of 1,254 complications in 21,258 patients (excluding 44 patients with LMWH plus ASS, 14 of them with complications)

The observed frequencies of adverse pregnancy outcome in 21,302 patients are demonstrated in Table 1. Table 2 describes the percentiles of the calculated probabilities of APO for the 21,302 patients. To get an impression of a possible effect of ASS alone or LMWH alone on the frequency of observed APO, we performed a LOESS—regression for all 1.254 complications in 21,258 patients (Fig. 1) after having excluded 44 patients with a combined medication of LMWH plus ASS. In patients without anticoagulant therapy, the figure shows the steadily ascending relation between calculated and observed frequencies of complication which is due to the construction of the cpAPO risk predictor. Both ASS and LMWH had a complication-reducing effect in patients at very high risk for APO. The effect was less pronounced and started in patients with higher cpAPO values in the LMWH group.

**Table 1:** Frequency of observed forms of APO in 21,302 patients

Complication	*n*	%
PD < 33	207	0.97
PE	297	1.39
PE < 33	39	0.18
IUGR ≤ P5	792	3.71
PA	52	0.24
IUD/NND	61	0.29
All	1,268	5.95

*PD* *<* *33* preterm delivery <33 gestational weeks, *PE* preeclampsia, *PE* *<* *33* preeclampsia delivered <33 weeks, *IUGR* *≤* *P5* intrauterine growth restriction at or below 5th percentile, *PA* placental abruption, *IUD/NND* intrauterine death/neonatal death

**Table 2:** Percentiles of calculated frequencies of complications in 21,302 patients

cpAPO	1.3 %	2.2 %	2.5 %	4.3 %	8.8 %	13.2 %	27.8 %	42.9 %	93.6 %
Percentile	min	5	10	50	90	95	98	99	Max

*cpAPO* Calculated probability of adverse pregnancy outcome

Anticoagulant therapy was started with ASS in 147 patients, with LMWH alone in 73 patients and with a combination of LMWH and ASS in 15 patients; 191 patients did not receive an anticoagulant therapy. None of the 426 patients of the high-risk subgroup had received anticoagulants during this pregnancy up to this time. BMI, age and cpAPO of the different groups of intervention are described in Table 3. The groups were comparable with the exception of a higher value of cpAPO in ASS patients in comparison to non-treated patients and in LMWH patients in comparison to ASS and non-treated patients.

**Table 3:** Descriptive statistics (median, range) for BMI, age, and cpAPO (calculated probability for adverse pregnancy outcome) for all 426 high-risk patients as well as the subgroups without and with anticoagulant therapy (ASS or LMWH or both, ASS only, LMWH only)

	All	No therapy	ASS only	LMWH only
	426 100 %	191 44.8 %	147 34.5 %	73 17.1 %
BMI	24.2 (16.7–46.4)	23.5 (16.7–42.5)	23.9 (17.2–44.1)	24.8 (18.2–46.4)
Age	32 (14–45)	32 (15–44)	32 (17–45)	33 (19–43)
CpAPO	42.9 (27.9–93.6)	40.6 (27.9–82.8)	43.0 (27.9–88.1) *p* = 0.035*	48.3 (29.0–93.6) *p* = 0.002**

* Result of Mann–Whitney’s *U* test versus no therapy

** Result of Mann–Whitney’s *U* test versus ASS

Table 4 gives an overview on the frequency of different forms of APO in all patients as well as patients with and without ASS and LMWH as well as the statistical analysis of differences of the frequency of all forms of APO. In all groups, the observed frequency of complications (39.3–45.1 %) exceeded the predicted minimal frequency of 27.8 % (P 98). The effect of ASS in comparison to patients without anticoagulants was a significant reduction of IUD/NND, PD <33 weeks and PE < 33 weeks as well as a non-significant tendency of lower frequencies of PA as well as PE. The frequency of IUGR was slightly higher with no statistical significance. The subgroup with LMWH treatment starting with a higher calculated probability for APO in comparison to the ASS and non-treated group did not show a significant reduction of any form of APO; in contrary, for PE and for the sum of all complications, there was a significant increase.

**Table 4:** Absolute and relative frequencies of complications in high-risk patients in dependence on no treatment as well as ASS or LMWH treatment

Complication	All (*n* = 426)	No therapy (*n* = 191)	ASS only (*n* = 147)	*P*	LMWH only (*n* = 73)	*P*
IUGR	82 (19.3 %)	30 (15.7 %)	35 (23.8 %)	0.061 (↑) n.s.	13 (17.8 %)	n.s.
IUD/NND	10 (2.3 %)	10 (5.2 %)	0 (0 %)	0.006 ↓	0 (0 %)	0.066 (↓) n.s.
PA	15 (3.5 %)	8 (4.2 %)	2 (1.4 %)	n.s.	4 (5.5 %)	n.s.
PD < 33	56 (13.1 %)	32 (16.8 %)	11 (7.5 %)	0.011 ↓	12 (16.4 %)	n.s.
PE	84 (19.7 %)	38 (19.9 %)	19 (12.9 %)	n.s.	24 (32.9 %)	0.026 ↑
PE < 33	29 (6.8 %)	18 (9.4 %)	4 (2.7 %)	0.013 ↓	6 (8.2 %)	n.s.
All complications	181 (42.5 %)	75 (39.3 %)	5 8(39.5 %)	n.s	40 (54.8 %)	0.023 ↑

Please note that several types of complications could occur in parallel. Result of statistical test (*χ* ^2^ test) for comparison of fractions of patients with complications between ASS treatment and non-treatment as well as LMWH treatment and non-treatment (↑ elevated fraction, ↓ decreased fraction)

*PD* *<* *33* preterm delivery < 33 gestational weeks, *PE* preeclampsia, *PE* *<* *33* preeclampsia delivered < 33 weeks, *IUGR* *≤* *P5* intrauterine growth restriction at or below 5th percentile, *PA* placental abruption. *IUD/NND* intrauterine death/neonatal death

## Discussion

With a frequency of APO of 5.95 %, in sum, the whole study group a priori fulfilled the criteria of a “low” or “normal” risk group. Application of the algorithm for individual risk calculation showed the heterogeneity of this group consisting of patients with a range from a very low to a very high probability of APO (Table 2).

Table 3 shows the biological data as well as the calculated frequencies of APO in relation to non-medication as well as application of ASS and LMWH. While there was no significant difference between the groups concerning BMI and age, the cpAPO values of the three groups differed significantly. With a cpAPO value of 43.0, the ASS group had a significantly higher a priori probability in comparison to the non-treated group (cpAPO of 40.6 %). With a cpAPO value of 48.3, the LMWH group had a significantly higher a priori probability for APO in comparison to both the ASS group as well as the non-treated group. These differences may—at least partly—contribute to the results shown in Table 4. With an observed frequency of 39.5 % in ASS patients, there was no significant difference to the observed frequency of 39.3 % in patients without treatment. Regarding the different forms of APO, in spite of the higher a priori probability of APO in the ASS group, there was a significant complication-reducing effect of ASS for IUD/NND, PD < 33 and PE < 33. These data confirm prior reports [26–37] showing a complication-reducing effect of ASS in high-risk patients. In addition, the data also show that the protecting effect of ASS even is present if the administration starts in the second half of pregnancy.

In contrast, there was a—non significant (*P* = 0.061)—increasing effect of ASS on the frequency of IUGR. While the majority of studies focus on the influence of ASS on PE, the observation of the influence of ASS on IUGR is limited [36, 49]. The tendency of higher frequencies of IUGR in our retrospective evaluation may be partly explained by a pregnancy-prolonging effect of ASS which decreases the frequency of the severest complications PD < 33, PE < 33 and IUD/NND perhaps inducing the effect of a higher frequency of (surviving) IUGR babies. It also has to be kept in mind that the a priori probability in the ASS group was significantly higher than that in the non-treated group. In addition, the late beginning of administration of ASS may play a role in the elevated frequency of IUGR in ASS patients. In a meta-analysis of 27 studies, it was shown that the reducing effect of ASS on IUGR was only seen if administration started before 16 weeks [36].

There is evidence that the complication-reducing effect is restricted to high-risk patients. In low-risk patients, ASS was not effective in reducing the risk [30]. The method of identifying high-risk patients may be a reason why some studies did find only moderate effect of ASS or even no effect at all [26, 28, 29]. Our method based on biophysical and sonographic data only has proved to be effective in identifying patients at high risk for APO [3]. In addition, it has the advantage of being cost-effective. The more affordable and practicable a method of prediction is, the greater is the chance of increasing the number of patients who might potentially take advantage from prediction and following therapeutics. From an ethical perspective which takes questions of justice and allocation into account, one might argue that it would be desirable to give access to prediction and treatment of APO to as many women as possible which is facilitated if the method is cheap, simple, safe, and fast. The additional measurement of serum parameters only in patients identified by this algorithm to be at high risk may increase cost-effectiveness in comparison to a concept of primary serum parameter measurement in all patients.

There is little evidence that also Heparin and LMWH alone or in combination with ASS [41, 50, 51] may have beneficial effects in high-risk pregnancies to prevent PE and IUGR [52]. Some observational as well as randomised [51] studies have shown a protective, complication-reducing effect of LMWH in thrombophilic [41, 43] as well as in non-thrombophilic patients [42, 44]. But not all studies could show a protective effect of heparin [52, 53]. The evidence showing the potential of LMWH to prevent recurrence of PE is limited, so the use of Heparin and LMWH is considered to be experimental [54]. Our retrospective evaluation could not show a complication-reducing effect of LMWH administration if started in the second half of pregnancy. In contrast to ASS, our 73 patients treated with LMWH did not show a significant reduction or mitigation of any form of APO. This may partly be explained by some of the shortcomings of the study: it was not prospective double-blinded, but retrospective; there was a significantly higher median of cpAPO especially in patients treated with LMWH, so the LMWH group contained patients with a significantly higher risk of APO. Another factor may be the smaller number of patients of the LMWH group. And it cannot be excluded that LMWH has a protecting effect only if administration starts early in pregnancy.

There are several more shortcomings of the study that have to be mentioned. This retrospective evaluation covers a long time period in which also other measures of treatment like intensity of survey of high-risk pregnancies or the quality of neonatal care may have changed. The algorithm applied here is based on few parameters and does not regard important factors like patient’s history, pre-existing morbidity like hypertension, thrombophilia or other relevant maternal diseases.

A crucial point is the indication of anticoagulants. During the time of this retrospective analysis, we did not have an algorithm to quantify the risk for APO, so there was no clear basis for indication and mode of intervention with the consequence of intervention on a subjective arbitrary basis. Also the situation of published evidence appeared to shift from a preferential administration of ASS to a more positive view of the impact of LMWH. The mode of interventions changed with time with a higher frequency of patients treated with ASS at the beginning of the observation and a change to a higher frequency of application of LMWH in the later years. The patients were primarily surveyed by their gynecologist and sent to our center for the ultrasound examination. The decision whether and which anticoagulant was administered often based on subjective parameters. So besides objective factors like allergy against ASS, there were also reasons like antipathy of the patient against injections or refusal of the referring physician to administer expensive drugs. We cannot exclude that other unknown confounders might have influenced the results.

These findings suggest further research on the question as to which patients had most benefit from which alternative prophylactic medication. However, prospective, randomized trials in pregnant women face severe ethical problems. Above all, researchers fear danger for the fetus. Experiences of teratogenic effects of administered drugs and other dangers for mother and fetus through, for example, placenta crossing drugs have resulted in ethics guidelines restricting research with pregnant women [55, 56]. Pregnant women are often excluded from research, and ethicists start to claim that research within this group has become a “moral imperative”, because “if a population is going to use a medication, it must be studied in that population” [57]. At the same time, these authors warn that risk–benefit trade off between woman and fetus need to be managed by several safeguards. One of these safeguards includes the pursuit of innovative study designs. We argue that retrospective research designs serve the purpose of reducing risks by preselecting rational and reasonable hypotheses for further prospective studies that have a low-risk profile and are beneficial for the study participants. Retrospective data offer indispensable information about the safety, effectiveness, and efficiency of interventions.

Our data show that also the administration of anticoagulants starting in the second half of pregnancy is of advantage for high-risk patients. As a consequence, we raise doubt that regarding the body of evidence concerning ASS it may be justified not to administer anticoagulants in high-risk patients or to perform studies of ASS against placebo.

With regard to the evaluation of our data, the effectiveness of a medication of LMWH beginning in the second half of pregnancy stays unclear. At least, we could not show a beneficial effect of LMWH—which is much more expensive than ASS—beginning at the second half of pregnancy. To clear the situation, a prospective study—perhaps ASS alone against LMWH as well as a combination of both—might be helpful.
